# Progress in the Mechanism of Heat Shock Proteins in Gastric Cancer: From Biomarkers to Therapeutic Targets

**DOI:** 10.1002/iid3.70542

**Published:** 2026-10-19

**Authors:** Wang Yanan, Zhang Jinpeng, Zhou Peng, Wang Yuan, Wang Qingyi, Liang Guoying

**Affiliations:** ^1^ The First Clinical Medical College of Heilongjiang University of Chinese Medicine Harbin Heilongjiang China; ^2^ The Second Affiliated Hospital of Heilongjiang University of Chinese Medicine Harbin Heilongjiang China; ^3^ The First Affiliated Hospital of Heilongjiang University of Chinese Medicine Harbin Heilongjiang China

**Keywords:** biomarkers, heat shock proteins, stomach neoplasms, therapeutic targets, tumor

## Abstract

**Background:**

Gastric cancer remains a major challenge in public health, with low early diagnosis rates and poor prognosis for advanced patients. Heat shock proteins (HSPs) are highly conserved molecular chaperones that maintain proteostasis and promote cell survival under stress. Aberrantly elevated HSPs in gastric cancer are closely linked to tumor initiation, invasion, metastasis, and poor prognosis. This review aims to comprehensively synthesize the latest advances concerning HSPs in gastric cancer across three core dimensions: their utility as diagnostic/prognostic biomarkers, their regulatory mechanisms in key signaling pathways and the tumor microenvironment (TME), and their viability as therapeutic targets.

**Materials and Methods:**

This review is structured as a narrative synthesis of the literature. A structured search was performed across PubMed, EMBASE, Web of Science Core Collection, and Cochrane Library, from their inception to March 2026. The search strategy combined keywords and MeSH terms related to three domains: (1) HSPs; (2) gastric cancer; and (3) therapeutics/inhibitors. Two authors independently screened titles and abstracts, and disagreements were resolved through discussion or consultation with a third author. Observational human studies, experimental studies (in vitro and in vivo), and preclinical animal models relevant to the three thematic pillars were included. Reference lists of included papers and relevant review articles were also manually searched. Given the broad scope and qualitative, integrative nature of this review, no formal quantitative meta‐analysis or standardized risk‐of‐bias assessment was performed. Instead, findings were synthesized thematically, emphasizing critical appraisal of conflicting data, identification of knowledge gaps, and discussion of translational barriers.

**Results:**

HSPs exhibit heterogeneous expression in gastric cancer tissues, which is associated with tumor differentiation, lymph node metastasis, and progression. HSP70 expression correlates with poor differentiation, high invasiveness, intestinal‐type gastric cancer, and lymphovascular invasion, and is involved in proliferation, migration, anti‐apoptosis, and chemoresistance; inhibition of HSP70 enhances therapeutic sensitivity. HSP90ab1 interacts with LRP5, inhibits its ubiquitin‐mediated degradation, activates AKT and Wnt/β‐catenin signaling, and promotes epithelial–mesenchymal transition and invasive metastasis. Plasma HSP90α is elevated in gastric cancer patients and correlates with CEA, CA125, CA153, CA199, and immune cells, as well as with metastatic stage. HSP27 is elevated in gastric cancer tissues, promotes tumor growth by inhibiting PANoptosis, and is associated with progression and poor prognosis. HSPs also participate in TME remodeling by regulating cancer‐associated fibroblasts, immune cells, vascular endothelial cells, and the extracellular matrix. They can both activate anti‐tumor immunity and promote immunosuppression and immune evasion, and are involved in multidrug resistance. Mechanistically, HSPs interact with pathways such as PI3K/Akt, MAPK, and TGF‐β, showing context‐dependent and biphasic regulatory roles. HSP inhibitors, especially HSP90 inhibitors such as ganetespib, have shown preclinical and clinical activity in gastric cancer, but single‐agent efficacy is limited by toxicity, poor tumor penetration, and HSF1‐driven compensatory upregulation of HSP70, HSP40, and HSP27. Combination with chemotherapy, immunotherapy, photothermal/photodynamic therapy, and nanocarrier‐based delivery has shown potential to improve efficacy. Dual targeting of HSP70/HSF1 and individualized combination regimens are proposed future directions.

**Discussion:**

HSPs are key molecules in the initiation and progression of gastric cancer. Their abnormal expression reflects tumor biological characteristics and provides new avenues for clinical diagnosis and treatment. The mechanisms of HSPs in gastric cancer are complex, involving stress responses, protein folding/repair, signaling pathway regulation, and immune evasion, which determine their critical roles in the TME. As biomarkers, HSPs have shown sensitivity and specificity in early diagnosis and prognosis, but clinical translation still faces challenges such as inhibitor selection, protection of normal cells, and drug resistance. Future research requires multidisciplinary collaboration to resolve existing controversies, clarify molecular mechanisms, and develop efficient and safe targeted drugs, so as to improve the diagnosis and treatment outcomes of gastric cancer and enhance patient survival and quality of life.

## 1 Introduction

Gastric cancer, one of the malignant tumors with high incidence and mortality rates worldwide, remains a major challenge to be addressed urgently in the field of public health [1]. Although the diagnosis rate of early gastric cancer has improved in some regions (e.g., Europe) in recent years—through enhanced awareness of gastric cancer risk, popularization of high‐resolution endoscopy, and application of virtual chromoendoscopy—thereby improving survival rates, the overall early diagnosis rate of gastric cancer is still low. A high proportion of patients are diagnosed at an advanced stage, resulting in limited treatment options and poor prognosis [2]. The development of gastric cancer is closely associated with multiple factors. It shows significant geographical variations, with particularly high incidence in East Asia. Meanwhile, Helicobacter pylori infection, dietary habits, genetic factors, and environmental exposure are all recognized as important pathogenic factors [3]. Currently, the diagnosis of gastric cancer mainly relies on upper gastrointestinal endoscopy and tissue biopsy. Moreover, the symptoms of early gastric cancer are mostly atypical, which further increases the difficulty of early detection [4].

Heat shock proteins (HSPs), as core molecules in cellular stress responses, have received extensive attention in cancer research in recent years. HSPs are a class of highly conserved molecular chaperones. They mainly maintain intracellular proteostasis by assisting protein folding, repairing misfolded proteins, and promoting the degradation of irreversible proteins, thereby helping cells resist various internal and external environmental stresses, including heat shock, oxidative stress, and drug stimulation [5, 6]. In gastric cancer and other malignant tumors, HSPs usually exhibit high expression, which helps tumor cells evade apoptosis, promote cell proliferation and invasion, and thus drive tumor initiation and progression [7]. Heat shock factor 1 (HSF1), the core regulator of HSP expression, not only plays a key role in stress responses but also its excessive activation in various cancers is closely related to malignant tumor progression and poor prognosis [8]. Studies have found that HSPs not only contribute to the survival and protection of gastric cancer cells but also enhance tumor invasiveness and metastatic potential by regulating cellular metabolic reprogramming, promoting epithelial–mesenchymal transition (EMT), and maintaining stemness [9].

Based on the important role of HSPs in gastric cancer, researchers have recently explored their potential as biomarkers and therapeutic targets. The expression levels of certain HSPs are closely related to patients' clinical stages and prognosis, and can be used for auxiliary diagnosis and risk stratification [10]. Inhibitors targeting HSPs and their regulatory factors—such as ganetespib targeting HSP90—have shown promising application prospects in the treatment of various cancers and have gradually entered the clinical trial stage [11]. HSPs interact with tumor‐related signaling pathways, immune microenvironment, and cell death pathways (e.g., PANoptosis) to influence tumor biological behavior, which also provides new ideas for precision therapy targeting HSPs. However, the dynamic regulatory mechanisms of HSPs and their specific roles in different molecular subtypes of gastric cancer still require further in‐depth research.

Gastric cancer, as a malignant tumor with high mortality, still faces great challenges in early diagnosis and effective treatment. As key regulators of cellular stress responses, HSPs play an important role in the initiation, progression, and treatment of gastric cancer. Systematically sorting out the molecular mechanisms of HSPs in gastric cancer and their clinical application value will help advance the progress of early gastric cancer diagnostic technologies and innovate targeted therapy strategies, thereby improving patients' survival prognosis and quality of life. This article will focus on the latest research progress of HSPs in gastric cancer, deeply explore their potential as biomarkers and therapeutic targets, and provide a theoretical basis and research direction for future precision diagnosis and treatment of gastric cancer.

## 2 Materials and Methods

This review is structured as a narrative synthesis of the literature, aiming to provide a comprehensive and critical overview of the roles of heat shock proteins in gastric cancer—from molecular mechanisms to therapeutic applications.

Literature Search: To ensure broad and reproducible coverage of the evidence, we performed a structured search across four major biomedical databases: PubMed, EMBASE, Web of Science Core Collection, and Cochrane Library, from their inception to March 2026. The search strategy combined keywords and MeSH terms related to three domains: (1) HSPs (“Heat Shock Proteins,” “HSP,” and “Heat Shock”); (2) gastric cancer (“Gastric,” “Stomach,” “Cardia,” “Cancer,” “Neoplasm,” and “Tumor”); and (3) therapeutics (“inhibitor”). The core Boolean logic was: (Heat Shock Proteins OR HSP OR Heat Shock) AND (Gastric OR Stomach OR Cardia) AND (Cancer OR Neoplasm OR Tumor) AND (inhibitor OR its synonyms). No language or publication date restrictions were imposed.

Study Selection: Two authors independently screened the titles and abstracts of all retrieved records. Disagreements were resolved through discussion or, if necessary, by consultation with a third author. Full texts of potentially relevant articles were then assessed for inclusion based on their relevance to the three thematic pillars of this review: (1) expression patterns and clinical significance of HSPs in gastric cancer; (2) their regulatory roles in the tumor microenvironment and key signaling pathways (e.g., PI3K/Akt and MAPK); and (3) therapeutic targeting, including preclinical and clinical trial evidence. We included observational human studies (cohort, case‐control, and cross‐sectional), experimental studies (in vitro and in vivo), and preclinical animal models. In addition, we manually searched the reference lists of included papers and relevant review articles to identify any further studies.

Synthesis Approach: Given the broad scope and the qualitative, integrative nature of this review, we did not perform a formal quantitative meta‐analysis or a systematic risk‐of‐bias assessment using standardized tools. Instead, we synthesized the findings thematically, grouping evidence according to the above three pillars. Our synthesis emphasizes critical appraisal of conflicting data, identification of knowledge gaps, and discussion of translational barriers.

## 3 Classification and Functions of Heat Shock Proteins (HSPs)

### 3.1 Key Roles in Cellular Homeostasis and Disease

HSPs are a family of highly conserved molecular chaperones that are universally expressed across all organisms. They play pivotal roles in protein folding and unfolding, assembly of multi‐protein complexes, trafficking and sorting of proteins to correct subcellular compartments, cell cycle regulation, signal transduction, as well as protection of cells against stress and apoptosis [12]. Based on differences in molecular weight and function, HSPs are primarily classified into several families. The most representative ones include the HSP70, HSP90, and HSP60 families, along with small heat shock proteins (sHSPs) such as HSP27 [13, 14]. Additionally, the HSP superfamily encompasses the HSP40 and HSP100 families [15, 16].

#### 3.1.1 The HSP70 Family

As one of the most crucial molecular chaperones, the HSP70 family is involved in the folding of newly synthesized polypeptides, assembly of protein complexes, and refolding of denatured proteins under stress conditions, with its activity dependent on ATP [17]. Beyond its key role in maintaining cellular proteostasis, HSP70 also participates in cell signal transduction, cell cycle regulation, and apoptosis [18]. Acting as a co‐chaperone for HSP90, HSP70 contributes to the stabilization of lysosomal membranes and is involved in apoptotic body formation and cellular senescence [19]. Under stress, HSP70 binds to damaged proteins to facilitate their refolding or degradation, thereby protecting cells from the detrimental effects of misfolded proteins [20]. In tumor biology, overexpression of HSP70 is closely associated with the proliferation, migration, and anti‐apoptotic capabilities of cancer cells, and it has demonstrated emerging research value in the field of immuno‐oncology [21]. Meanwhile, the Hsp70 gene exhibits selective overexpression in human tumors, which correlates with poor prognosis, resistance to chemotherapy and radiotherapy, and advanced tumor stages [22].

#### 3.1.2 The HSP90 Family

The HSP90 family primarily functions in the stabilization and activation of mature proteins, particularly in the folding and functional maintenance of tumor‐associated proteins [23]. In addition to its role in proteostasis, HSP90 regulates immune responses in the tumor microenvironment, making it a critical target for cancer therapy [24]. Within the tumor microenvironment, HSP90 acts in concert with other HSPs (e.g., HSP72 and HSP70). This collaboration can either enhance the immune system's recognition of tumor cells or promote tumor cell survival, proliferation, and immune evasion [25]. Given its close association with the development of various tumors, inhibitors targeting HSP90 have become a research focus in anti‐cancer drug development [26].

#### 3.1.3 The HSP60 Family

HSP60 is mainly localized in mitochondria, where it serves as a key chaperone for mitochondrial protein folding and maintains the homeostasis of mitochondrial function [27]. Studies have shown that HSP60 not only participates in intracellular protein folding but also is linked to apoptosis, immune responses, and the pathogenesis of various diseases [28]. Abnormal expression of HSP60 has been reported in inflammation, cardiovascular diseases, and tumors, indicating its multiple roles in pathophysiological processes [29]. Intracellularly, HSP60 interacts with glutathione and E‐cadherin, exhibits antioxidant properties, and helps protect mitochondria from oxidative damage [30]. Research has demonstrated that silencing of HSP60 leads to the loss of simian immunodeficiency virus (SIV)‐mediated cellular protection, increased mitochondrial apoptosis, and even destabilization or enhanced stability of p53, thereby promoting apoptosis [19].

#### 3.1.4 Small Heat Shock Proteins (sHSPs)

Small heat shock proteins, which include HSP10, HSP20, HSP27, and HSP32 based on molecular weight, are ATP‐independent “holdase” chaperones. They primarily assist cells in coping with adverse conditions such as heat shock by binding to unfolded or partially folded proteins and preventing their aggregation [31]. HSP27 exerts protective effects in various tumors and cardiovascular diseases, and its expression level is usually associated with disease progression [32].

#### 3.1.5 The HSP40 and HSP100 Families

The HSP40 family acts as co‐chaperones for HSP70, aiding in protein folding, substrate delivery, and regulation of stress responses. Although direct evidence linking HSP40 to gastric cancer is limited, its synergistic interaction with HSP70 suggests that it may indirectly promote gastric cancer progression by enhancing HSP70‐mediated cellular protection [33]. The HSP100 family is mainly involved in protein disaggregation and stress tolerance in prokaryotes and eukaryotes. Its role in gastric cancer remains poorly studied, but its ability to mediate stress adaptation implies that it may be involved in the survival of gastric cancer cells under harsh microenvironmental conditions [34].

In summary, the HSPs superfamily comprises multiple important members that play key roles in maintaining cellular proteostasis, regulating cellular functions, and responding to various stresses. As core components of cellular protective mechanisms, HSPs have become research hotspots for biomarkers and therapeutic targets due to their significant roles in multiple diseases, especially tumors, cardiovascular diseases, and neurodegenerative diseases. Understanding the structure, function, and regulatory mechanisms of different HSP types is of great significance for further elucidating their mechanisms of action in gastric cancer and other diseases. Figure 1 presents a summary of the formation and function of HSPs.

**FIGURE 1:**
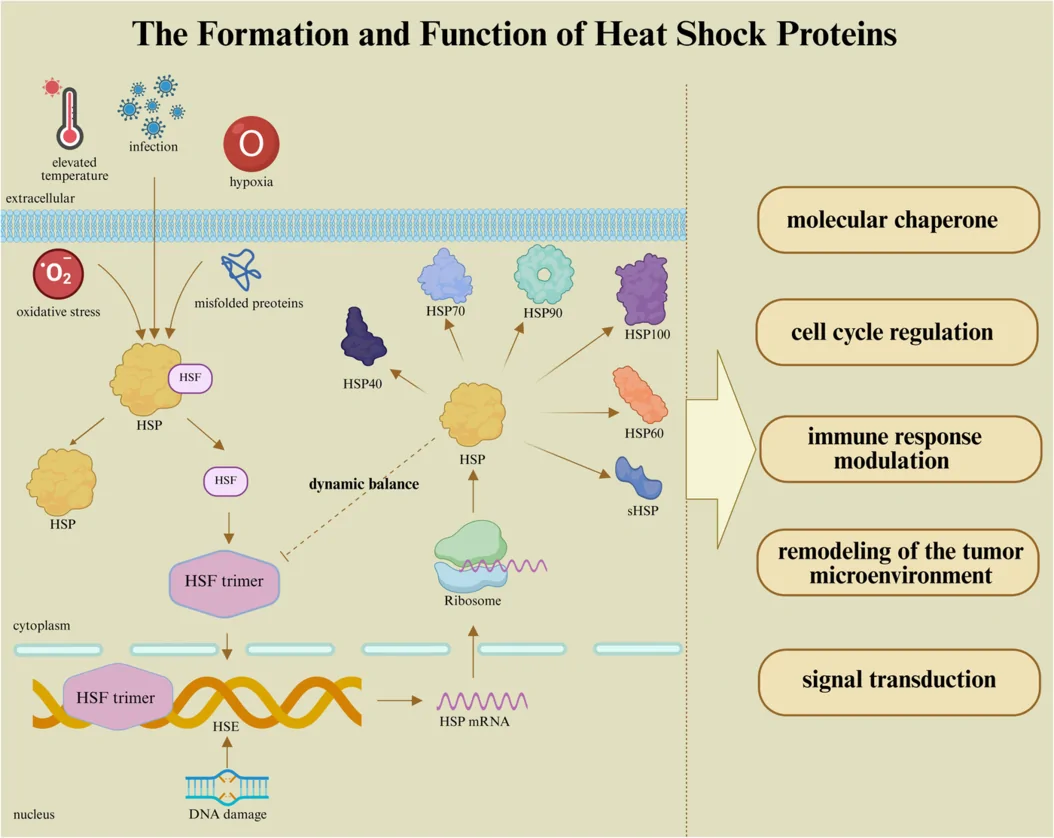
The formation and function of heat shock proteins. Heat shock proteins (HSPs) are a highly conserved family of intracellular proteins whose expression is significantly upregulated in response to cellular stresses (e.g., heat shock, oxidative stress, hypoxia, and toxic substance exposure). This stress‐induced synthesis is mainly regulated by heat shock factors (HSFs), which bind to heat shock elements (HSEs) in HSP gene promoters after activation, initiating transcription and translation. Functionally, HSPs act as molecular chaperones to facilitate correct folding of newly synthesized polypeptides, prevent aggregation of misfolded/denatured proteins, and mediate degradation of irreversibly damaged proteins via the ubiquitin–proteasome system. Beyond chaperone activity, HSPs participate in critical cellular processes (cell cycle regulation, apoptosis, immune response modulation, and proteostasis maintenance) and are involved in gastric cancer pathogenesis by regulating stress tolerance, signal transduction, and TME remodeling.

### 3.2 Role in Cellular Stress Responses With Emphasis on Gastric Cancer

HSPs enable cells to survive environmental stresses (heat, oxidative stress, and chemotherapeutic agents) through their chaperone functions, including protein folding, refolding, and clearance of misfolded proteins [35, 36, 37, 38]. In gastric cancer, this cytoprotective machinery is hijacked to promote chemoresistance and survival under hostile conditions. For instance, HSP70 upregulates the anti‐apoptotic protein Bcl‐2 and inhibits the activities of pro‐apoptotic proteins Bax and caspase‐3, thereby blocking intrinsic apoptosis in gastric cancer cells exposed to cisplatin or 5‐fluorouracil [39, 40, 41]. Similarly, HSP90 interacts with the autophagic receptor NBR1 to regulate selective autophagy, a process that helps gastric cancer cells tolerate nutrient deprivation and hypoxic stress within the tumor microenvironment [42].

The heat shock response is coordinated by HSF1, which in gastric cancer is constitutively activated and drives HSP overexpression. HSF1 not only binds to heat shock elements in HSP gene promoters but also regulates satellite DNA‐associated lncRNAs, which further modify stress‑response gene expression programs in gastric cancer cells [43]. During the recovery phase from stress, HSP70‑dependent restoration of protein solubility is essential for gastric cancer cell survival; inhibition of HSP70 delays recovery and increases chemosensitivity [44]. Moreover, the microRNA pathway participates in turning off the heat shock transcriptional program after stress, ensuring cell survival [45]. These stress‑adaptive mechanisms are markedly more active in gastric cancer than in normal gastric mucosa, providing a potential therapeutic window for HSP inhibitors.

In summary, in the context of gastric cancer, HSPs act as key enablers of stress tolerance, apoptosis resistance, and metabolic adaptation, making them attractive targets for intervention. The above content is summarized in Table 1.

**TABLE 1:** Classification, molecular functions of heat shock protein (HSP) families and their mechanisms of action in gastric cancer.

HSP family	Representative members	Core molecular functions	Mechanisms of action in gastric cancer	References
HSP70	HSP70, HSP72	ATP‐dependent assistance in folding of newly synthesized polypeptides and refolding of denatured proteins; Regulation of cell apoptosis, signal transduction, and autophagy; Acting as a co‐chaperone of HSP90 to stabilize lysosomal membranes	High expression promotes proliferation, migration, and anti‐apoptosis of tumor cells; Binding to Smad3 to inhibit TGF‐β‐induced early EMT; Participation in the “ROS‐JNK‐HSPs” negative feedback loop to enhance chemoresistance	[17, 18, 19, 20, 21, 22, 41, 46, 47]
HSP90	HSP90ab1, HSP90AA1	Stabilization of the structure and function of mature proteins (especially oncoproteins such as c‐MYC and c‐MET); Regulation of immune responses in the tumor microenvironment (TME); Interaction with NBR1 to participate in selective autophagy	Inhibition of ubiquitin‐mediated degradation of LRP5, activation of the AKT/Wnt/β‐catenin pathway to promote EMT; Formation of a complex with alpha‐fetoprotein (AFP) to drive tumor progression; Stabilization of TGF‐β receptors to enhance late‐stage tumor metastasis	[23, 24, 25, 26, 48, 49, 50, 51, 52]
HSP60	HSP60	Localized in mitochondria, maintaining mitochondrial protein folding and functional homeostasis; Binding to glutathione to exert antioxidant effects; Regulation of apoptosis and immune responses	Abnormal expression associated with tumorigenesis; Induction of mitochondrial apoptosis after silencing, affecting p53 stability; Potential regulation of tumor metabolism through mitochondrial function modulation	[19, 27, 28, 29, 30]
Small HSPs	HSP27, HSP20	ATP‐independent “holdase” to prevent protein aggregation	HSP27 inhibiting PANoptosis to promote tumor growth, inducing secretion of IL‐10/TNF‐α to enhance immune evasion; HSP20 inhibiting the MAPK/AKT pathway to reduce tumor invasiveness	[31, 32, 53, 54, 55, 56, 57]
HSP40	HSP40	HSP40 acting as a co‐chaperone of HSP70 to assist protein folding; Regulation of cellular stress tolerance and signal pathways	HSP40 indirectly promoting tumor progression through synergy with HSP70	[33]
HSP100	HSP100	Mediation of protein disaggregation and stress adaptation; Participation in stress tolerance in prokaryotes and eukaryotes	May support the survival of gastric cancer cells in hypoxic and nutrient‐poor microenvironments through stress adaptation mechanisms; specific mechanisms remain to be elucidated	[34]

## 4 Expression and Clinical Significance of Heat Shock Proteins in Gastric Cancer

HSPs exhibit expression levels in gastric cancer tissues that are closely associated with the clinicopathological characteristics of tumors, making them one of the critical focuses in current gastric cancer research. Studies have demonstrated significant heterogeneity in HSP expression within gastric cancer tissues; this heterogeneity not only reflects the diversity of tumor cells but also correlates tightly with tumor differentiation grade, lymph node metastasis, and tumor progression [58]. Single‐cell transcriptomic analysis using patient‐derived organoid (PDO) models has revealed marked differences in HSP expression among gastric cancer organoids from different anatomical sites. These differences modulate the tumor immune microenvironment, particularly the activity of cytotoxic T cells, suggesting a pivotal role of HSPs in regulating anti‐tumor immune responses [59].

### 4.1 HSP70: A Mediator of Malignant Progression and Therapeutic Resistance

As a core member of the HSP family, HSP70 expression correlates closely with gastric cancer differentiation and lymph node metastasis. High HSP70 expression is significantly elevated in gastric cancer tissues with poor differentiation and high invasiveness, implying its potential role in promoting the malignant progression of tumor cells [58]. A systematic review and meta‐analysis indicated that high HSP70 expression is strongly associated with tumor differentiation grade, intestinal‐type gastric cancer, and lymphovascular invasion, but shows no significant correlation with overall patient survival. Additionally, upregulated HSP70 expression is linked to gastric cancer cell resistance to hyperthermic chemotherapy and apatinib; inhibiting HSP70 can enhance therapeutic sensitivity, highlighting its functional relevance in chemotherapy [60].

### 4.2 HSP90: Regulating Metastasis and Serving as a Diagnostic Biomarker

HSP90, particularly its HSP90ab1 isoform, interacts with low‐density lipoprotein receptor‐related protein 5 (LRP5) to inhibit LRP5 ubiquitin‐mediated degradation. This interaction activates the AKT and Wnt/β‐catenin signaling pathways, promotes the EMT process, and thereby enhances the invasive and metastatic capabilities of gastric cancer cells [48]. Furthermore, plasma HSP90α levels are significantly increased in gastric cancer patients, showing positive correlations with tumor markers such as CEA, CA125, CA153, and CA199. It also correlates closely with immune cells (including T cells, B cells, and the Th/Ts ratio), indicating its potential role in tumor immune regulation [49]. Notably, HSP90α expression is closely associated with the metastatic stage of gastric cancer, suggesting its value as a potential biomarker for tumor metastasis and progression [50]. Although plasma HSP90 levels have limited utility in predicting the survival of gastric cancer patients, when used as an auxiliary diagnostic indicator in combination with immune cell parameters, they improve diagnostic sensitivity and specificity—outperforming the CEA/CA19‐9 panel in auxiliary diagnostic efficacy [61]. Additionally, HSP90 has been found to regulate the stability of tumor‐associated proteins (c‐MYC and c‐MET) in gastric cancer cells and promote tumor progression by forming a complex with alpha‐fetoprotein (AFP), providing a novel perspective for investigating the molecular mechanisms of gastric cancer and identifying therapeutic targets [51].

### 4.3 HSP27: Inhibiting Programmed Cell Death and Predicting Prognosis

HSP27, another extensively studied HSP family member, shows elevated expression in gastric cancer tissues and associated oral squamous cell carcinoma—particularly in high‐risk populations—indicating its potential role in cancer development [53]. HSP27 promotes tumor growth by inhibiting PANoptosis (a form of programmed cell death), providing a theoretical basis for its use as a prognostic biomarker and therapeutic target [54]. The above content is summarized in Table 2.

**TABLE 2:** Expression characteristics and clinical significance of heat shock proteins (HSPs) in gastric cancer.

HSP family member	Expression characteristics	Clinically relevant indicators	Biomarker potential	References
HSP70	Significantly increased expression in poorly differentiated and highly invasive gastric cancer tissues; Higher expression in precancerous lesions than in normal gastric mucosa	Associated with tumor differentiation (increased risk of poor differentiation) and intestinal‐type gastric cancer; Correlated with lymphovascular invasion, but no direct correlation with lymph node metastasis; No significant association with overall patient survival	Auxiliary diagnosis of tumor malignancy, distinguishing between high/low invasiveness subtypes; Prediction of chemoresistance (to 5‐fluorouracil, cisplatin, apatinib); Potential target for resistance reversal therapy	[41, 58, 60]
HSP90	High expression of HSP90ab1 in gastric cancer tissues; Significantly higher plasma HSP90α levels in patients than in healthy individuals	Plasma HSP90α positively correlated with tumor markers such as CEA, CA125, CA153, and CA199; Closely associated with lymph node metastasis stage (higher expression in stage Ⅲ/Ⅳ); Correlated with T cell, B cell activity, and Th/Ts ratio	Limited diagnostic sensitivity (approximately 65%) when used alone as a diagnostic marker; Combination with immune cell parameters can improve diagnostic specificity to 82%, outperforming the CEA/CA199 panel; Indication of tumor metastasis risk, guiding postoperative follow‐up strategies	[48, 49, 50, 61]
HSP27	Expression level in gastric cancer tissues 2–3 times higher than that in normal tissues; Synchronously high expression in oral squamous cell carcinoma (high‐risk population for gastric cancer)	Associated with tumor progression (T stage) and poor prognosis; 37% increased recurrence risk in patients with high expression; Correlated with immunosuppressive microenvironment (increased Tregs infiltration)	Potential prognostic marker for independent prediction of disease‐free survival; Provides a target for therapies targeting the PANoptosis pathway; May serve as a screening indicator for high‐risk populations of gastric cancer	[53, 54, 55]
HSP90AA1	Regulated by lncRNA ZNRD1‐AS1, with high expression in gastric cancer tissues; Associated with cancer stem cell‐like properties	Correlated with tumor proliferation index (Ki‐67 positive rate); Positively correlated with the risk of distant metastasis (e.g., liver metastasis)	Serves as a downstream effector molecule of the lncRNA ZNRD1‐AS1/miR‐9‐5p axis, indicating directions for targeted therapy; Prediction of tumor recurrence and distant metastasis potential	[52]

## 5 The Role of Heat Shock Proteins in the Tumor Microenvironment

HSPs play a crucial regulatory role in the tumor microenvironment (TME), promoting cancer initiation and progression—particularly by modulating interactions between different cell types within the TME to drive tumor advancement. Beyond their canonical function as molecular chaperones that facilitate protein folding and stabilization, HSPs regulate the functions of tumor‐associated cells. They are involved in orchestrating immune responses, inflammatory reactions, cell migration, angiogenesis, and drug resistance mechanisms, thereby influencing the biological behavior of tumors and their response to therapy.

### 5.1 HSP‐Mediated Regulation of Tumor‐Associated Fibroblasts

Cancer‐associated fibroblasts (CAFs) are key components of the TME that promote tumor progression. Studies have shown that HSF1—the transcription factor governing HSP expression—is activated in gastric cancer‐derived CAFs. HSF1 regulates the expression of genes such as inhibin subunit beta A and thrombospondin 2; the proteins encoded by these genes are released into the TME via extracellular vesicles secreted by CAFs, enhancing the malignant phenotype and invasive capacity of tumor cells [62]. This finding indicates that HSPs and their regulatory factor HSF1 modulate intercellular communication in the TME by influencing the secretory function of CAFs, thereby promoting the invasion and metastasis of gastric cancer.

### 5.2 Dual Roles of HSPs in Modulating the Tumor Immune Microenvironment

HSPs exert dual effects on the regulation of the tumor immune microenvironment. On one hand, exosomes released by tumor cells contain HSP70; these exosomes can act as damage‐associated molecular patterns (DAMPs) to activate immune cells and enhance anti‐tumor immune responses [63]. On the other hand, overexpressed HSPs can induce an immunosuppressive microenvironment—for example, by promoting the polarization of regulatory T cells (Tregs) and M2‐type macrophages, and inhibiting the activity of effector immune cells—thereby facilitating immune evasion [64]. Additionally, HSP70 interacts with transforming growth factor‐β (TGF‐β) in the TME to regulate angiogenesis, suppress anti‐tumor immunity, and promote tumor progression [65].

### 5.3 HSPs in Tumor Therapeutic Resistance

In the context of tumor therapeutic resistance, HSPs drive the development of multidrug resistance (MDR) by regulating inflammatory pathways and immune checkpoint pathways. Specifically, HSP27 induces the secretion of immunosuppressive cytokines (e.g., IL‐10 and TNF‐α), impairs the differentiation of monocytes into mature dendritic cells, and promotes the formation of tumor‐associated macrophages. These effects collectively enhance tumor immune evasion and chemoresistance [55]. Thus, HSPs are not only critical molecules enabling tumor cells to adapt to TME stress but also key regulators of the tumor immune microenvironment.

### 5.4 HSP‐Based Therapeutic Strategies and Drug Delivery

Progress has also been made in developing HSP‐targeted therapies and drug delivery systems. Nanoparticles constructed using small‐molecule HSPs can respond to the acidic pH and overexpressed enzymes in the TME, enabling targeted drug release. This approach improves the efficacy of anti‐tumor therapy while reducing damage to normal tissues [66]. Concurrently, inhibiting the expression or function of HSPs can enhance the sensitivity of tumors to radiotherapy, chemotherapy, and immunotherapy, thereby improving treatment outcomes [67].

In summary, HSPs promote tumor growth, invasion, metastasis, and therapeutic resistance by regulating interactions between various cell types in the TME—including CAFs and immune cells. Gaining a deeper understanding of the mechanisms underlying HSP function in the TME will facilitate the development of novel diagnostic and therapeutic strategies targeting HSPs and their regulatory networks, ultimately improving the treatment efficacy for malignant tumors such as gastric cancer and enhancing patient survival rates. The above content is summarized in Table 3.

**TABLE 3:** Regulatory roles of heat shock proteins (HSPs) in the tumor microenvironment (TME) of gastric cancer.

TME‐component cells/structures	HSP‐mediated regulatory mechanisms	Impact on gastric cancer progression	References
Cancer‐associated fibroblasts (CAFs)	HSF1 activation in CAFs, regulation of INHBA and THBS2 gene expression; Secretion of extracellular vesicles containing HSP‐related proteins	Enhancement of malignant phenotypes of tumor cells (30% increase in invasiveness); Promotion of tumor stromal remodeling, acceleration of metastasis	[62, 68]
Immune cells (T cells, macrophages)	HSP70 in tumor‐derived exosomes activating dendritic cells (DCs) to enhance anti‐tumor immunity; HSP27 inducing IL‐10 secretion to promote M2 macrophage polarization; HSP90α inhibiting CD8 + T cell activity	Dual regulation: both activation of anti‐tumor immunity and promotion of immune evasion; 22% reduction in immunotherapy response rate in patients with high HSP expression	[49, 55, 63, 64]
Vascular endothelial cells	Interaction between HSP70 and TGF‐β to promote angiogenesis; HSP90 stabilizing VEGF receptors to enhance angiogenic signaling	40% increase in tumor microvessel density, acceleration of nutrient supply; Association with tumor metastasis (increased risk of vascular invasion)	[65]
Extracellular matrix (ECM)	HSP47 regulating collagen synthesis and deposition; HSP90 promoting matrix metalloproteinase (MMP9) activity	Increased ECM stiffness, promotion of tumor cell invasion; Enhanced MMP9 activity, acceleration of ECM degradation and metastasis	[33, 48]

## 6 Interplay Between Heat Shock Proteins and Signaling Pathways in Gastric Cancer

HSPs, a class of highly conserved molecular chaperones, not only serve protective roles in cellular stress responses but also critically regulate tumor cell proliferation, migration, and survival through interactions with multiple signaling pathways, making them a major focus in cancer research. In the context of gastric carcinogenesis and progression, the interplay between HSPs and key signaling pathways, such as PI3K/Akt and MAPK, provides novel insights into the molecular mechanisms of gastric cancer and the identification of potential therapeutic targets.

Among the HSP family, HSP70 is one of the most extensively studied members and is widely involved in regulating cell proliferation and migration. Studies in hepatocellular carcinoma models have indicated that HSP70 promotes cell migration via activation of the AKT signaling pathway [69]. This finding suggests that HSP70 may enhance the proliferative and invasive capabilities of gastric cancer cells through the PI3K/Akt pathway. The PI3K/Akt pathway is a central regulator of cell proliferation and survival, and its aberrant activation is a key oncogenic mechanism in various cancers. The molecular chaperone function of HSP70 may stabilize key proteins within the PI3K/Akt pathway, thereby facilitating signal transduction and driving the malignant phenotype of gastric cancer cells [70, 71]. Inhibition of HSP70 has been shown to restore the sensitivity of gastric cancer cells to 5‐fluorouracil (5‐FU) and cisplatin, identifying HSP70 as a crucial protective factor in cisplatin resistance [41]. The heat shock response can inhibit TGF‐β‐induced EMT through HSP70 binding to Smad3 and preventing its nuclear translocation. Conversely, in advanced tumors, HSP90 stabilizes the TGF‐β receptor, enhances Smad3 activity, and promotes metastasis—illustrating a context‐dependent, biphasic regulatory role (“early inhibition, late promotion”) [46]. Furthermore, ROS induced by Helicobacter pylori infection or chemotherapeutic agents can activate JNK1/2, leading to upregulated HSP70/90 expression. The elevated HSPs, in turn, inhibit JNK overactivation‐induced apoptosis, forming a “ROS‐JNK‐HSPs” negative feedback loop that promotes gastric cancer cell survival under stress conditions [47].

The small heat shock protein HSP20 has also been demonstrated to inhibit the MAPK and AKT signaling pathways in tumors. In breast cancer cells, HSP20 overexpression significantly suppressed the phosphorylation of AKT, ERK, JNK, and p38, consequently inhibiting cell proliferation and migration [56]. Although this study focused on breast cancer, its mechanistic insights provide a reference for understanding HSP interactions with the MAPK pathway in gastric cancer. The MAPK signaling pathway, including ERK, p38, and JNK branches, is a crucial regulator of cell growth, differentiation, and migration. By modulating the activation status of the MAPK pathway, HSPs are likely involved in regulating the malignant behavior of gastric cancer cells. Additionally, HSP27 was found to participate in the PDGFRβ‐mediated PI3K/Akt and p38 MAPK signaling pathways in a spinal morphological tolerance model [57]. This implies that HSP27 may act as a signaling node, bridging receptor tyrosine kinases with downstream pathways to modulate adaptive responses and migration in tumor cells. Similar mechanisms might operate in gastric cancer cells, influencing tumor progression.

The regulation of signaling pathways in gastric cancer cells is complex and diverse. Research indicates that the gastric cancer‐associated factor EEF1A2 interacts with the heat shock protein HSPB8, promoting cell proliferation and migration [68]. Moreover, the gastric cancer‐associated lncRNA ZNRD1‐AS1 regulates the miR‐9‐5p/HSP90AA1 axis, influencing the proliferation and metastasis of gastric cancer cells, suggesting HSP90's involvement in signaling pathway regulation via microRNA mechanisms [52].

HSF1, the master regulator of HSP expression, is also intricately linked to signaling pathways. In gastric cancer‐associated cancer‐associated fibroblasts (CAFs), HSF1 regulates downstream factors and influences the tumor microenvironment via the secretion of extracellular vesicles, thereby promoting tumor progression [62, 68]. Furthermore, in gastric cancer cells, HSP90 can function as a signaling hub, modulating multiple pathways to support cancer cell survival and migration.

In summary, heat shock proteins orchestrate complex interactions with key signaling pathways such as PI3K/Akt and MAPK, driving the malignant transformation of gastric mucosa and disease progression. Mechanistically, HSP90 stabilizes client oncoproteins like AKT, c‐MET, and TGF‐β receptors, while HSP70 can facilitate AKT phosphorylation, creating a hyperactive signaling hub that promotes uncontrolled proliferation, epithelial–mesenchymal transition, and survival under stress. This HSP‐mediated pathway hyperactivation is a hallmark of aggressive gastric cancer phenotypes, including enhanced invasion and metastasis. However, the clinical success of HSP inhibitors is hindered by the remarkable plasticity of this signaling network. A critical challenge is the feedback activation of HSF1 upon pharmacological inhibition of HSP90. This HSF1‐driven compensatory response paradoxically upregulates other pro‐survival chaperones, notably HSP70 and HSP27, which can bypass the blocked pathway and restore malignant signaling through parallel routes, such as the MAPK cascade. Furthermore, the complexity is amplified by context‐dependent signaling crosstalk, where the role of a single chaperone may shift from tumor‐suppressive (e.g., HSP70 inhibition of TGF‐β‐induced early EMT) to tumor‐promoting (e.g., HSP90 stabilization of late‐stage TGF‐β signaling) depending on the tumor stage. This dynamic interplay not only fosters acquired resistance but also explains the lack of sustained efficacy observed with single‐agent HSP90 inhibitors in gastric cancer trials, as outcomes may be improved by rational combinatorial strategies that co‐target multiple nodes (e.g., HSP70 and HSF1) to overcome these adaptive survival mechanisms. Future research should therefore prioritize dissecting these complex, stage‐specific regulatory loops and developing combination regimens that anticipate and block compensatory signaling to achieve durable therapeutic responses.

## 7 Prospects of Heat Shock Proteins as Therapeutic Targets

### 7.1 Development and Application of HSP Inhibitors

Recent years have witnessed significant progress in the research on heat shock protein inhibitors for the treatment of gastric cancer. In tumor cells, HSPs can protect various oncoproteins from degradation, thereby promoting tumor growth, invasion, and drug resistance. Consequently, targeting HSPs has become a key focus in anti‐cancer therapy, with HSP90 inhibitors, in particular, showing broad application prospects in various cancers.

In gastric cancer, studies have found that high HSP90 expression is closely associated with tumor progression and poor prognosis. The long non‐coding RNA ZNRD1‐AS1 promotes the proliferation and metastasis of gastric cancer cells by regulating the miR‐9‐5p/HSP90AA1 axis; knocking down ZNRD1‐AS1 significantly suppresses gastric cancer development, underscoring the critical role of HSP90 in this context [52]. Inhibitors targeting HSP90, such as ganetespib, have demonstrated anti‐cancer activity in various tumors, including gastric cancer. Ganetespib inhibits HSP90 function, promoting cancer cell apoptosis and growth arrest. Compared to other HSP90 inhibitors, it exhibits lower side effects and is undergoing multiple clinical trials, highlighting its potential value as a novel therapeutic agent for gastric cancer [11].

Regarding clinical trials, HSP90 inhibitors like 17‐AAG (17‐allylamino‐17‐demethoxygeldanamycin) have been investigated in studies for various cancers. Although specific clinical trial data for gastric cancer are still limited, existing evidence indicates that HSP90 inhibitors can improve tumor sensitivity to treatment and reduce the development of drug resistance. 17‐AAG inhibits HSP90, protects blood‐brain barrier integrity, and diminishes the capacity of tumor cells to tolerate therapy—a mechanism that may also be applicable to gastric cancer treatment [72]. Furthermore, comprehensive treatment strategies combining HSP inhibitors with other therapies, such as photothermal therapy or chemotherapeutic agents, are under development. These approaches leverage HSP inhibitors to reduce the tolerance of tumor cells to thermal stress, thereby enhancing therapeutic efficacy [73].

The development of novel nanocarrier systems also provides technical support for the application of HSP inhibitors. For instance, PEG‐modified phospholipid micelles co‐loading quercetin (a natural HSP inhibitor) and a photothermal agent can target tumors via nanotechnology, enhance drug endocytosis, and improve anti‐tumor effects. This strategy has shown promising therapeutic potential in breast cancer and other solid tumors and could potentially be extended to gastric cancer treatment in the future [74].

In summary, research on HSP inhibitors, especially HSP90 inhibitors, in gastric cancer therapy is advancing rapidly. By inhibiting the stress protection mechanisms of tumor cells, promoting cancer cell apoptosis, and enhancing the efficacy of conventional treatments, they hold considerable promise for clinical application. Future research should further elucidate their specific mechanisms of action in gastric cancer and optimize combination treatment regimens to facilitate their clinical translation and widespread use.

### 7.2 Combination Therapy Strategies Targeting Heat Shock Proteins

Currently, the therapeutic efficacy of single‐agent HSP‐targeted therapy is limited. In recent years, a growing number of studies have focused on the combined application of HSP inhibitors with other treatment modalities (such as chemotherapy, immunotherapy, photothermal therapy, etc.), aiming to exert synergistic effects and improve the treatment response rate and survival time of gastric cancer patients.

First, the combination of HSP inhibitors and chemotherapy has shown significant potential. As chaperones for various tumor‐related proteins, HSP90 inhibitors can not only induce tumor cell apoptosis but also enhance the sensitivity to chemotherapeutic drugs. A study demonstrated that the combination of HSP90 inhibitors such as 17‐DMAG with the targeted drug lapatinib can effectively overcome drug resistance in breast cancer cells, a strategy that also holds application potential in gastric cancer [75]. Additionally, the combination of HSP70 inhibitors and HSF1 inhibitors has exhibited synergistic antitumor activity, and this dual‐target blocking strategy is expected to overcome the limitations of negative feedback mechanisms associated with single‐agent therapy [61, 76].

Second, the combination of HSP inhibitors and immunotherapy provides a new approach for gastric cancer immunotherapy. HSP90 inhibitors not only disrupt key oncogenic proteins within tumor cells but also regulate the tumor microenvironment, enhancing the efficacy of immune checkpoint inhibitors (such as PD‐1/PD‐L1 antibodies). A study showed that NDNB1182, a specific inhibitor of HSP90β, can enhance the effectiveness of immune checkpoint blockade therapy and improve the outcome of immunotherapy‐refractory solid tumors [77].

Furthermore, the combination of HSP‐targeted photodynamic therapy (PDT) with immune checkpoint inhibitors can significantly enhance the antitumor immune response and prolong survival [78]. In photothermal therapy (PTT) and PDT, tumor cell tolerance to thermal stimulation mainly relies on the overexpression of HSPs. To overcome this barrier, researchers have designed various nanoplatforms integrating HSP inhibitors with photothermal or photodynamic agents, enabling simultaneous HSP inhibition and tumor cell killing. A study found that nanocarriers loaded with HSP90 inhibitors (such as SNX2112 nanomicelles) can significantly downregulate HSP90 expression and enhance the efficacy of PTT/PDT [79]. Meanwhile, nanosystems combining starvation therapy agents such as glucose oxidase indirectly inhibit HSP expression by reducing ATP supply, further improving photothermal/photodynamic therapeutic effects [80].

In addition, the combination of HSP inhibitors with multimodal therapy is also being actively explored. For example, the integration of mild photothermal therapy, chemotherapy, and catalytic therapy using nanocomposites achieves HSP downregulation and enhances antitumor efficacy [81]. This strategy substantially increases tumor cell sensitivity to treatment by reducing the protective effect of HSPs.

Notably, combination therapy strategies still face numerous challenges in clinical application, including drug dosage optimization, toxicity management, patient selection, and the development of biomarkers. Several clinical trials are currently evaluating the combined application of HSP90 inhibitors with other treatment modalities, and some, such as pimitespib combined with immune checkpoint inhibitors, have shown favorable tolerability and efficacy [82].

In conclusion, combination therapy strategies involving HSP inhibition, through multi‐target and multi‐mechanism synergistic effects, are expected to overcome issues of drug resistance and limited efficacy in gastric cancer treatment. In the future, personalized combination therapy regimens based on molecular subtyping and immune microenvironment characteristics will be crucial for improving the prognosis and survival rate of gastric cancer patients. With the development of nanotechnology, gene editing, and multi‐omics technologies, the precise regulation of combined HSP targeting with other therapies will further promote the advancement of comprehensive gastric cancer treatment. To provide a comprehensive overview, Table 4 summarizes the major preclinical and early‑stage therapeutic strategies targeting HSPs in gastric cancer, including single‑agent inhibitors, combination regimens, and nanocarrier‑based approaches. Table 5 complements this by presenting representative completed and ongoing clinical trials, highlighting both successes (pimitespib) and failures (ganetespib), as well as early‑phase exploratory studies.

**TABLE 4:** HSP‐targeted therapeutic strategies and research progress in gastric cancer.

Therapeutic type	Targeted target/pathway	Specific strategy	Research stage	Efficacy and safety characteristics	References
Single‐target inhibitors	HSP90	Ganetespib (small‐molecule inhibitor); NDNB1182 (HSP90β‐specific inhibitor)	Ganetespib: Phase Ⅱ clinical trial (gastric cancer indication); NDNB1182: Preclinical research	Ganetespib: Up to 28% ORR in some cancer types; in gastric cancer phase II trial, ORR 4% (1/26), trial terminated early due to insufficient efficacy; NDNB1182: Enhancement of PD‐1 antibody efficacy, reversal of immune resistance	[11, 77]
Combination with chemotherapy	HSP70 + chemotherapeutic agents	Quercetin (HSP70 inhibitor) + cisplatin; HSP70 inhibitor + 5‐fluorouracil	Preclinical research (cell and animal models)	Reversal of cisplatin resistance, 40% increase in tumor inhibition rate; 30% reduction in 5‐fluorouracil dosage without compromising efficacy	[41, 74]
Combination with immunotherapy	HSP90 + immune checkpoint inhibitors	Ganetespib + PD‐1 antibody (e.g., pembrolizumab); NDNB1182 + PD‐L1 antibody	Ganetespib: Phase Ⅰb clinical trial; NDNB1182: Preclinical research	ORR of combination therapy 15%–20% higher than that of monotherapy; Reduction of Tregs infiltration, increase in cytotoxic T cell (CD8+) recruitment	[77, 78]
Nanocarrier‐mediated therapy	HSP inhibitors + photothermal/photodynamic agents	PEG‐phospholipid micelles (loaded with quercetin + photothermal agent); SNX2112 nanomicelles (HSP90 inhibitor + PDT agent)	Preclinical research (mouse gastric cancer model)	5‐fold increase in tumor targeting, reduction of normal tissue toxicity; Photothermal therapy combined with HSP inhibition, tumor ablation rate reaching 90%	[66, 74, 79]
Dual‐target inhibition	HSP70 + HSF1	HSP70 inhibitor + HSF1 inhibitor (e.g., KRIBB11)	Preclinical research	Blockade of the “HSP70‐HSF1” negative feedback loop, 25% higher tumor growth inhibition rate than monotherapy	[61, 76]

**TABLE 5:** Representative clinical trials of HSP‑targeted therapies in gastric cancer.

Agent/drug	Primary target	Phase	Gastric cancer enrollment	Key results & safety	References
Ganetespib (STA‐9090)	HSP90	II (NCT01167114)	28 patients with advanced esophagogastric cancer (≤ 2 prior lines)	Negative: ORR 4% (1/26); median OS 83 days (≈2.8 mo). Trial terminated early. AEs: diarrhea (77%), fatigue (65%).	[83]
Minnelide + paclitaxel	HSP70 (via Sp1 inhibition)	I (2021–2022)	23 refractory metastatic gastric cancer; 20 received ≥ 3 lines	Exploratory positive: In 11 evaluable patients: 1 PR, 5 SD (2 SD > 6 mo). No DLT. Well tolerated.	[84]
Pimitespib + nivolumab	HSP90 + PD‐1	Ib (EPOC1704)	8 gastric cancer patients (total 44 solid tumors)	Feasibility demonstrated: Safe; in MSS colorectal cancer (ICI‑naïve) ORR 16%; pimitespib reduced Treg activity.	[85]
AUY922 (Luminespib)	HSP90	Preclinical + supportive data	Preclinical gastric cancer models	Preclinically positive: Induces apoptosis, inhibits invasion; suppresses YAP1/TEAD, enhances CD8^+^ T cell function.	[86]

Abbreviations: AEs, adverse events; DLT, dose‑limiting toxicity; ICI, immune checkpoint inhibitor; MSS, microsatellite stable; ORR, objective response rate; OS, overall survival; PR, partial response; SD, stable disease; Treg, regulatory T cell.

Despite encouraging preclinical data, the clinical translation of HSP inhibitors in gastrointestinal cancers, including gastric cancer, has encountered significant hurdles that warrant a critical appraisal. First, the narrow therapeutic index of first‐generation inhibitors, such as geldanamycin derivatives, is largely attributed to dose‐limiting toxicities, notably hepatotoxicity and gastrointestinal distress, which restrict the achievement of effective systemic concentrations [87, 88]. Second, the physicochemical properties and pharmacokinetic profiles of many HSP90 inhibitors lead to poor spatial penetration and heterogeneous distribution within solid tumor masses, resulting in suboptimal target inhibition in deep‐seated or poorly vascularized tumor regions [89]. Third, and perhaps most critically, tumors often engage robust compensatory survival pathways upon HSP inhibition. For instance, the pharmacological blockade of HSP90 triggers a rapid and potent heat shock response, driven by HSF1, leading to a counterproductive upregulation of HSP70, HSP40, and HSP27. This compensatory chaperone network can effectively buffer the stress induced by the drug, preserving the function of alternative oncogenic clients and enabling the cancer cells to adapt and survive [90]. Therefore, the failure of agents like ganetespib in phase II trials for gastric cancer can be viewed not as a failure of the target itself, but as a testament to the remarkable adaptability of the cancer stress response. Acknowledging these barriers is crucial for guiding future research toward more effective strategies, such as the design of isoform‐selective inhibitors with improved safety profiles, the use of advanced drug delivery systems to enhance tumor penetration, and the rational development of combinatorial regimens that co‐target the compensatory HSF1‐HSP70 axis to prevent resistance from the outset.

Collectively, the clinical evidence reveals a heterogeneous landscape: some HSP90 inhibitors (e.g., ganetespib) failed due to lack of efficacy, and HSP70‑directed agents remain in early phases. Key lessons include the importance of drug class (second‑generation inhibitors with better pharmacokinetics and tolerability), patient selection (late‑line, heavily pretreated populations may benefit from cytostatic rather than cytotoxic mechanisms), and combination strategies (immunotherapy and chemotherapy). Future research should focus on: (1) identifying predictive biomarkers (e.g., baseline HSP expression levels, molecular subtypes) to enrich responsive patients; (2) optimizing rational combinations, especially with ICIs, through well‑designed Phase II/III trials; (3) developing third‑generation inhibitors with greater selectivity for cancer‑relevant HSP isoforms (e.g., HSP90α/β or tumor‑specific post‑translational modifications); and (4) exploring dual inhibition of HSP70 and HSF1 to overcome feedback activation.

## 8 Controversies and Future Directions

Despite significant progress in HSP research, controversies remain. First, HSPs exhibit dual roles in the TME—enhancing anti‐tumor immunity (e.g., HSP70 activating dendritic cells) and promoting immune evasion (e.g., HSP27 inducing Treg polarization). The mechanisms underlying this duality and their context dependence (e.g., tumor stage, molecular subtype) require further clarification.

Second, clinical translation of HSP biomarkers faces challenges. For example, plasma HSP90α has limited single‐marker diagnostic sensitivity (≈65%), and combination with other markers (e.g., immune cell parameters) needs validation in large multi‐center cohorts. Additionally, HSP expression heterogeneity among different gastric cancer subtypes (e.g., intestinal vs. diffuse type) requires subtype‐specific biomarker development.

Looking forward, we propose several highly specific translational next steps grounded in clinical and surgical realities. In the short term, we advocate for the incorporation of circulating HSP70 and HSP90α as dynamic pharmacodynamic biomarkers in prospective clinical trials to enable real‐time monitoring of treatment response and early detection of resistance. Concurrently, the evaluation of HSP inhibitor efficacy in patient‐derived organoid models from different Lauren subtypes (intestinal vs. diffuse) could help identify patient populations most likely to benefit, thereby informing future trial design. In the medium term, we recommend the design of adaptive clinical trials that co‐target the compensatory HSF1‐HSP70 axis from the outset, for example, by combining HSP90 inhibitors with a HSF1 inhibitor like KRIBB11. This strategy directly addresses the most critical mechanism of acquired resistance. Moreover, given the poor penetration of current inhibitors into solid tumors, we see a clear path for leveraging nanotechnology to develop novel formulations, such as tumor microenvironment‐responsive nanoparticles, that can deliver HSP inhibitors more efficiently and specifically to primary tumors and metastatic lesions, potentially reducing systemic toxicity. Long‐term, a multimodal approach will be essential. We envisage that the synergistic combination of HSP90 inhibitors with immune checkpoint inhibitors could be a game‐changer, and we propose that this combination be prioritized in clinical trials for microsatellite‐stable gastric cancers, a patient subgroup with an urgent unmet need. Finally, we underscore the importance of collaborative, multicenter biobanking initiatives that collect paired primary and metastatic tumor tissues, as well as longitudinal blood samples. These resources would be invaluable for dissecting the spatial and temporal heterogeneity of HSP expression and for identifying robust predictive biomarkers to guide patient selection and combination therapies in the next generation of clinical trials.

## 9 Conclusions

Heat shock proteins are key molecules in gastric cancer initiation and progression. Their abnormal expression reflects tumor biological characteristics and provides new avenues for clinical diagnosis and treatment. Studies have confirmed correlations between HSP expression and gastric cancer invasiveness, metastasis, and prognosis—offering insights into molecular mechanisms. HSP mechanisms in gastric cancer are complex, involving stress responses, protein folding/repair, signal pathway regulation, and immune evasion—collectively determining their critical TME roles. Current research has advanced understanding of HSP biological functions, but inconsistencies exist in specific mechanisms and clinical applications. For example, studies differ on whether HSPs promote progression via stress tolerance or protect via immune activation—highlighting the need to distinguish HSP roles across gastric cancer subtypes and stages for precision therapy. HSP potential as biomarkers has been validated in clinical studies, showing high sensitivity and specificity in early diagnosis and prognosis—improving early detection and guiding personalized treatment. However, clinical translation of HSP therapeutic targets faces challenges (e.g., inhibitor selection, normal cell protection, and drug resistance). Future research requires multidisciplinary collaboration (molecular biology, medicinal chemistry, and clinical trial design) to advance HSP‐targeted therapy from theory to practice.

In summary, HSPs have significant scientific and clinical value in gastric cancer research. Resolving existing controversies, clarifying molecular mechanisms, and developing efficient/safe targeted drugs will be future focuses. These efforts are expected to improve gastric cancer diagnosis and treatment outcomes, ultimately enhancing patient survival and quality of life.

## Author Contributions

**Wang Yanan:** formal analysis, conceptualization, writing – original draft, writing – review and editing, supervision. **Zhang Jinpeng:** investigation. **Zhou Peng:** writing – original draft, visualization, methodology. **Wang Yuan:** investigation. **Wang Qingyi:** investigation. **Liang Guoying:** writing – review and editing, funding acquisition, project administration.

## Ethics Statement

The authors have nothing to report.

## Conflicts of Interest

The authors declare no conflicts of interest.

## Data Availability

The data that support the findings of this study are available from the corresponding author upon reasonable request.
